# Dr. Wm. Langley

**Published:** 1916-09

**Authors:** 


**Dr. Wm. Langley,** not listed in Polk or State Directory, died at Angelica, July 22, aged 40.

